# Giant condyloma acuminatum–malignant transformation

**DOI:** 10.1002/ccr3.863

**Published:** 2017-02-23

**Authors:** Ioannis K. Papapanagiotou, Kyriaki Migklis, Georgia Ioannidou, Dimitra Xesfyngi, Vasileios Kalles, Theodoros Mariolis‐Sapsakos, Emmanouil Terzakis

**Affiliations:** ^1^ Department of GynecologyPeripheral Cancer Hospital “Agios Savvas”Alexandras ave. 171Athens11522Greece; ^2^ Department of SurgeryGeneral and Oncological Hospital of Kifissia “Agioi Anargyroi”University of AthensTimiou stavrou 14, KifissiaAthens14564Greece; ^3^ Department of RadiotherapyPeripheral Cancer Hospital “Agios Savvas”Alexandras ave. 171Athens11522Greece

**Keywords:** Anal mass, Buschke–Löwenstein tumor, giant condyloma acuminatum, malignant transformation

## Abstract

Giant condyloma acuminata are associated with malignant transformation in up to 50% of cases, high recurrence rate, and poor prognosis. Treatment strategies have included wide local excision, abdominopelvic resection, and addition of radiotherapy and adjuvant and/or neoadjuvant systemic chemotherapy.

Question‐Quiz: What is this condition and how should it be treated?

Answer: A 53‐year‐old woman visited our gynecology emergency room complaining about a perianal mass, bleeding, and odor. Clinical examination revealed a giant mass originating from anorectum. Biopsy samples of the tumor were retrieved and sent for pathological examination. Results indicated a well‐differentiated squamous cell carcinoma on the ground of a giant (12.5 cm horizontally × 16 cm transversally × 10.5 cm anteroposteriorly) condyloma acuminata (Fig. 1). Multidisciplinary oncological team indicated primarily radiation therapy, to stop bleeding and reduce the volume of the tumor, systemic chemotherapy, and prophylactic colostomy to prevent future bowel obstruction.

**Figure 1:**
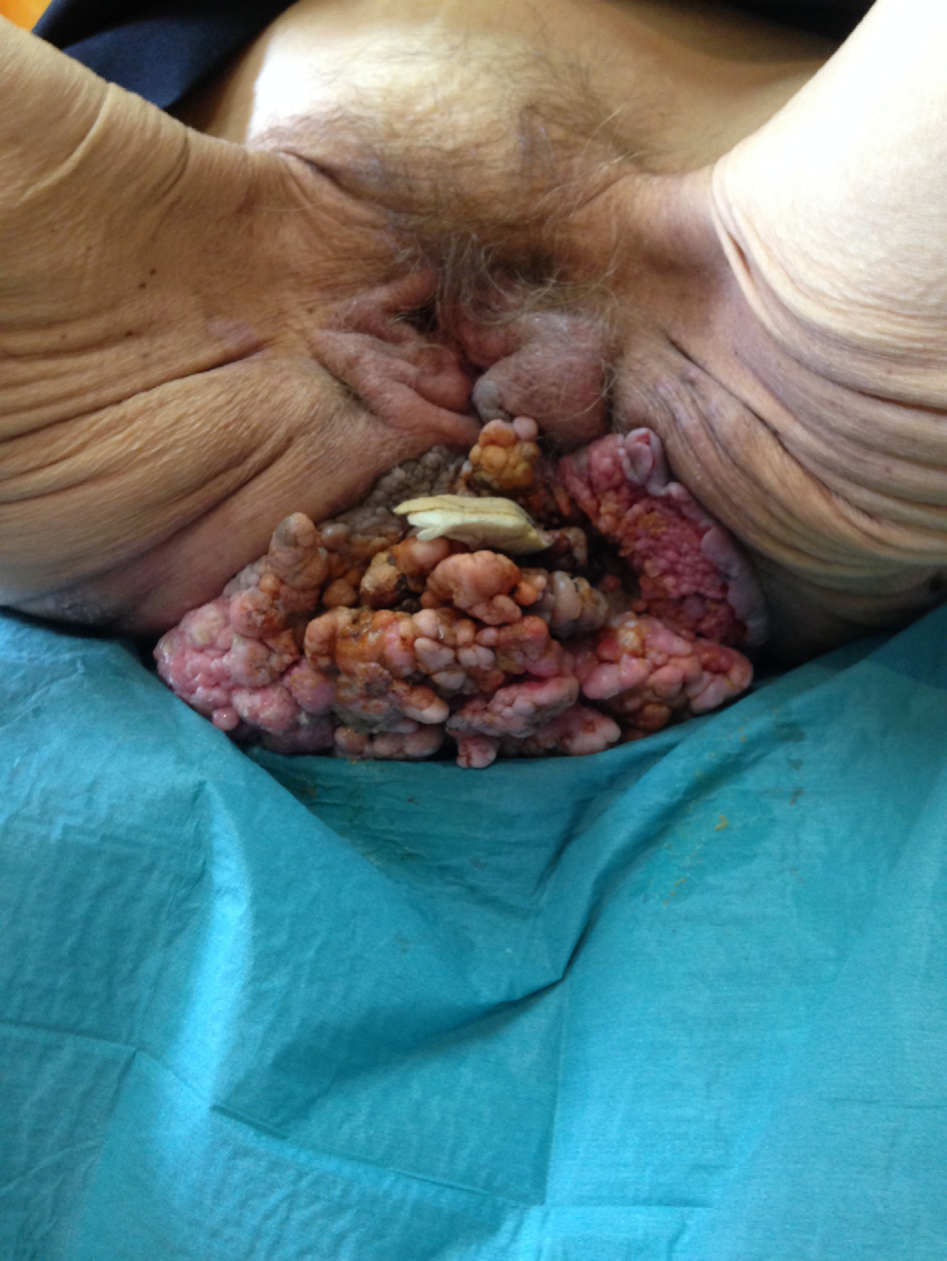
Giant condyloma acuminatum originating from anorectum.

## Authorship

IKP: Obstetrician and Gynaecologist, Author: involved in primary handling of the patient in the emergency room; KM: Obstetrician and Gynaecologist, Co‐author: involved in primary handling of the patient in the emergency room. GI: Oncology Radiologist, Member of the multidisciplinary oncological team: was responsible for radiation therapy received by the patient; DX: Oncology Radiologist: was responsible for radiation therapy received by the patient; VK: Surgeon, Member of the multidisciplinary oncological team: reviewed the manuscript; TMS: Surgeon, Director of the Surgical Department. ET: Obstetrician and Gynaecologist, Director of the Gynaecology Oncological Department. TMS and ET: were members of the multidisciplinary oncological team.

## Conflict of Interest

None declared.

